# Recent Developments in Mouse Trauma Research Models: A Mini-Review

**DOI:** 10.3389/fphys.2022.866617

**Published:** 2022-04-29

**Authors:** Adrian Gihring, Fabian Gärtner, Melanie Schirmer, Martin Wabitsch, Uwe Knippschild

**Affiliations:** ^1^ Department of General and Visceral Surgery, Surgery Center, Ulm University Medical Center, Ulm, Germany; ^2^ Division of Pediatric Endocrinology and Diabetes, Department of Pediatrics and Adolescent Medicine, Ulm University Medical Center, Ulm, Germany

**Keywords:** trauma, mouse model, clinical relevance, immune response, translatability

## Abstract

The urgency to investigate trauma in a controlled and reproducible environment rises since multiple trauma still account for the most deaths for people under the age of 45. The most common multiple trauma include head as well as blunt thorax trauma along with fractures. However, these trauma remain difficult to treat, partially because the molecular mechanisms that trigger the immediate immune response are not fully elucidated. To illuminate these mechanisms, investigators have used animal models, primarily mice as research subjects. This mini review aims to 1) emphasize the importance of the development of clinically relevant murine trauma research, 2) highlight and discuss the existing conflict between simulating clinically relevant situations and elucidating molecular mechanisms, 3) describe the advantages and disadvantages of established mouse trauma models developed to simulate clinically relevant situations, 4) summarize and list established mouse models in the field of trauma research developed to simulate clinically relevant situations.

## Introduction

Although advances were made in therapeutic treatment of patients suffering multiple trauma, it still remains one of the main causes for death in the population under 45 years (Kung et al., 2008; Norton and Kobusingye 2013). These kinds of injuries can be caused by falls, car accidents, explosions and most frequently result in thorax trauma, extremity fractures, muscle trauma and head injuries (Bardenheuer et al., 2000) affecting functionality of substantial organs like lung, heart, brain, and kidneys but also the hematologic system, the immune system and the endocrine system, potentially leading to multiple organ dysfunction syndrome (MODS) (Marshall, 2001). Regarding the lung, trauma could result in acute respiratory distress syndrome (ARDS), characterized by dyspnea, hypoxemia, cellular infiltrates as well as alveolar degradation (Bakowitz et al., 2012). Cardiovascular dysfunction is characterized by lower cardiac output and stroke volume (Wall et al., 2019), dysrhythmias including ventricular fibrillation, cardiac arrest, and wall motion disorders as well as ongoing hypotension (B. Weber et al., 2021a; B. Weber et al., 2021b). Neurological disorders are described by altered level of consciousness assessed by the Glasgow coma score (Villeneuve et al., 2016) and most likely caused by impaired cerebral blood flow, vasospasm or cerebral edema (Vella et al., 2017; Jha et al., 2019). Furthermore, the kidneys can be affected by trauma resulting in acute kidney injury (AKI) characterized by decreased renal perfusion and glomerular filtration rate (GFR) (Lai et al., 2016; Makris and Spanou 2016). Apart from that, trauma can also affect the hematological system leading to trauma-induced coagulopathy (TIC) specified by hypocoagulation in early-stage and hypercoagulation in later-stage, platelet dysfunction, and dysregulated fibrinolysis (Hayakawa 2017; Peng and Su 2017; Moore et al., 2021). Especially traumatic brain injury is highly associated to dysfunctions concerning the endocrine hormonal system manifesting by adrenal insufficiency, diabetes insipidus or hyponatremia (Bollerslev et al., 2013). Many aspects of the patient care remain heavily debated due to unknown underlying mechanisms with regard to the immediate immune response. This instant response can be described as systemic inflammatory response syndrome (SIRS) and the compensatory anti-inflammatory response syndrome (CARS), where both require attention to avoid secondary consequences of multiple organ failure (Osuchowski et al., 2006; Novotny et al., 2012), persistent inflammation, immunosuppression, and/or protein catabolism syndrome (PICS) (Mira et al., 2018). Even though the basic principles behind the triggered immune response are fairly known, molecular mechanisms after multiple trauma are extremely complex and not fully elucidated yet.

Within the last years, more trauma models including hemorrhagic shock as an additional factor were established as the handling of these patients is still complex and the underlying mechanisms are not fully elucidated yet (Bouglé et al., 2013). Hypovolemic hemorrhagic shock leads to insufficient oxygen delivery and consequently to cellular death. The subsequent release of damage-associated molecular patterns (DAMPs) triggers the innate immune response (Pantalone et al., 2021). In a murine polytrauma model, additional hemorrhagic shock strongly influenced the innate immune response by upregulation of myeloid leukocyte activation and differentiation, upregulation of cytokine secretion of interleukin 6 (IL-6), interleukin 1β (IL-1β) and tumor necrosis factor *α* (TNF-α), upregulation of genes involved neutrophil chemotaxis and cell adhesion as well as upregulation of toll-like receptor signaling pathway. Apart from that a downregulation of pathways involved in B- and T-cell activation was observed indicating a dysfunction of adaptive immune activation (Debler et al., 2021). Consequently, hemorrhagic shock further promotes uncontrolled innate immune response potentially leading to disbalanced cascade systems such as acute trauma-induced complementopathy or coagulopathy (Huber-Lang and Ward 2018).

The acute response to trauma-hemorrhage involves a complex interplay between the brain and the peripheral visceral organs. Trauma often leads to a disruption of the endocrine brain function including pituitary dysfunction, impaired antidiuretic hormone secretion, adrenal hormone reduction and altered secretion of thyrotropin consequently influencing the catecholamine surge, inflammatory response as well as sympathetic tone (Rachfalska et al., 2020). Apart from that, gut barrier integrity and loss of mucus layer caused by metabolic alterations related to glycolysis, amino acid biosynthesis, pento-phosphate pathway and mitochondrial ATP synthesis seem to be highly involved in the development of MODS after trauma-hemorrhage (Z. Li et al., 2019). The epithelial barrier failure of the gut leads to the systemic release of tissue injury factors *via* the mesenteric lymph (toxic mesenteric lymph) affecting and damaging multiple organs including spleenic and thymic immune cell apoptosis (Tiesi et al., 2013), shift of Treg to Th17 cell ratio in the mesenteric lymph node (Morishita et al., 2015), induction of cardiac contractile dysfunction (Lee et al., 2008) as well as promoting lung injury (Levy et al., 2012). Furthermore, it was shown that hemorrhagic shock leads to apoptosis of bone-marrow hematopoietic progenitor cells (Kumar et al., 2016). Remarkably, it was shown that stimulation of the vagus nerve, which represents the longest parasympathetic nerve connecting the central nervous system with visceral organs and the immune system, mitigates gut barrier dysfunction and prevents systemic organ damage (Levy et al., 2012; Levy et al., 2013; Morishita et al., 2015).

In this context the call for models to investigate the ongoing mechanisms after trauma increases. This need can be filled by utilizing animal models, which offer options for a reproducible and controllable environment.

However, the choice of a suitable trauma model highly depends on hypothesis and scientific questions and should be carefully revised as later modifications or adaptions regarding the trauma model might be difficult to implement and are often related to high effort or flawed data.

**FIGURE 1:**
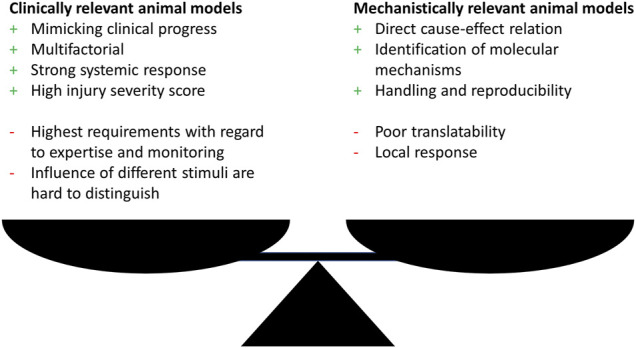
Advantages (+) and disadvantages (−) of clinically or mechanistically relevant animal trauma models.

Generally, research has utilized animal models that fall within one of the two categories: clinical relevance or elucidation of molecular mechanisms. Therefore, researchers should clarify whether the aim of the study is on illuminating a molecular mechanism possibly by using genetically modified organisms or on simulating a clinically relevant situation. Both approaches show an interdependency and choosing one approach often goes at the expense of the other one (Figure 1).

Nevertheless, either approach show drawbacks with the focus on translatability of the findings to the human situation. With the main focus in trauma research in mind, which inevitably is on developing animal models that simulate the clinical situation of complex response after trauma as close as possible, this might seem contradicting, since this represents the fundamental requirements for a successful transfer to the human. To overcome these shortcomings a third group of models is necessary, dealing with complex trauma settings, since in this research field relevant mechanisms can only be elucidated when the underlying trauma model was proven to be clinically relevant.

Complex trauma models, for example the combination of multiple trauma with hemorrhagic shock or infection, allow a systemic evaluation of the inflammatory response that is more important for the clinical progress but often lack the possibility to elucidate direct cause-effect relations and detailed molecular mechanisms due to multifactorial design (Figure 1). Although related to high additional effort, one approach to overcome these limitations might be the independent investigation of the single trauma as well as all possible trauma combinations as exemplarily performed by Denk et al. (2015) and Relja et al. (2020). The investigation of monotrauma should ideally be performed within the same study or at least under same conditions as the polytrauma. This strategy will greatly enhance the understanding of the complex trauma models and clarify how monotrauma contribute to the overall outcome. The establishment of models and the design of studies that combine both, clinical relevance, and the possibility to describe molecular mechanisms, should be the focus of further investigations.

Within recent years, this has been in the focus of researchers, who have developed new models and refined existing models by increasing complexity as well as severity of trauma with the aim to improve clinical translatability, which will be discussed in a later chapter (Table 1). This will include various murine animal models, that aim to add additional depth to their trauma model by advanced modifications closer to human conditions. These models try to diminish the negative aspects mentioned in Figure 1 as they are developed based on previous studies that investigated the effect of the respective single trauma (when applying multiple trauma) or they even address this issue in the presented study. Apart from that, the shown studies first describe and elucidate the impact of trauma on molecular, cellular, tissue, and organ level helping to evaluate whether the approach is useful to simulate a human clinical situation, illustrating an important aspect of the “reverse translation” approach introduced by Efron et al. (2015), discussed more detailed in chapter 3.

**TABLE 1:** Summary of trauma mouse models with a specific focus on clinical relevance by implementing additional aspects to add to translational clinical value.

Model	Perturbation	Implementation	Translational clinical value	Strain and age	Literature
Repetitive traumatic brain injury (rTBI)	Sudden rotation in coronal plane +	Custom-built device	Mimicking repeating head hits occurring in boxing or football	Male C57BL/6	Chen et al. (2019)
	Lateral translation		Human study:the Professional Fighters’ Brain Health Study Bernick et al. (2015)	12 weeks	
Polytrauma and shock	Hemorrhagic shock +	Injection of heparin +	Mimicking equivalent ISS of severely injured patients	n.a	Mira et al. (2018)
	Long bone fracture + Muscle tissue damage + cecectomy	cutting the tibia + bruise superior muscle +surgical removement of cecum	Human study: 5,761 trauma patients with open or closed femur fractures and shock Weber et al. (2016)	straight methodically description	
Combined blunt trauma	Blunt muscle trauma +	Weight drop device +	Mimicking multiple injuries from car accidents	Male C57BL/6	Gihring et al. (2020)
	Blunt thorax trauma	Single blast wave	Human study:110 polytrauma patients with blunt chest injury Chrysou et al., (2017)	16 ± 1 week	
Tibial fracture orthopedic injury model	Nociceptive sensitization +	Osteotomy with a micro drill +	Mimicking complex orthopedic injury after falls, motor vehicle crash or war-related injuries	Male C57BL/6J	Tawfik et al. (2020)
	bone fracture +	score the tibia with a bone saw causing trauma to tibialis anterior muscle +	Human study:approx. 28,000 patients with tibial shaft fracture Anandasivam et al. (2017)	13–17 weeks	
	muscle fibrosis +	complete bone fracture using counter pressure +			
	muscle fibre loss	intramedullary nail alignment			
Polytrauma with hemorrhagic shock	Blunt chest trauma +	Single blast wave +	Mimicking hemodynamically instable polytrauma with an injury severity score of at least 25	C57BL/6	Denk et al. (2018)
	Traumatic brain injury +	Weight drop device (333 g, 2 cm) +	Human study: 29 polytrauma patients with hemorrhagic shock (ISS 36 ± 11) Corradi et al. (2011)	8–9 weeks	
	closed transverse femoral fracture (inclusive soft tissue injury) +	Weight drop device (50 g, 120 cm) +			
	hemorrhagic shock	pressure-controlled blood drawing			
Blast-induced mild traumatic brain injury	Mild traumatic brain injury	Explosion device (500 g TNT) resulting in direct shockwave and reflected wave from the ground	Mimicking complex mild blast injury provoked by an explosive blast	Male ICR	Ratliff et al. (2020)
			Human study: 51 blast-exposed veterans with mild TBI Clark et al. (2018)	n.a.	
Abdominal trauma	Traumatic injury of the epigastrum	Single blast wave	Mimicking organ injury pattern induced by blunt abdominal after car or bike crash, child maltreatment or war-related injuries	Male C57BL/6JRj	Maitz et al. (2021)
			Human study: 99 patients with blunt abdominal injury Ntundu et al. (2019)	8–12 weeks	
Multiple trauma	Hemorrhagic shock +	Bled to blood pressure of 35 ± 5 mm Hg for 90 min +	Mimicking early post-traumatic inflammatory response in human	Male C57BL/6NCrl	Relja et al. (2020)
	thoracic trauma +	weight drop (500 g) induced plunger +	Human study: 54 patients suffering from multiple trauma combined with ALI Liang et al. (2016)	12 weeks	
	osteotomy +	saw the femur +			
	bilaterial soft tissue trauma +	weight drop (40 g) induced plunger +			
	laparotomy	2 cm midline laparatomy			

## Trauma Models With a Focus on Clinically Relevant Modifications

Various mouse models were defined and established over the last century. One of the first animal models in scientific literature was established by R. L. Noble and J. B. Collip in 1942 focusing on graded levels of trauma and their association with mortality curves, with a focus on complications of hemorrhage, infections as well as anesthesia (Noble and Collip 1942). In the context of this research the Noble-Collip drum was established, which has been used in trauma models since then (Moulton et al., 1962; Li et al., 2017; Hayakawa et al., 2015). Based on this, researchers came up with their own ways to set reproducible trauma in animal models including single as well as a combination of trauma. Some of the most recent mouse models with the aim to mimic the human condition of a traumatic injury are listed in Table 1, including one of our own murine models consisting of a combined blunt trauma (muscle + lung) using 16 ± 1 week old C57BL/6 mice (Gihring et al., 2020). The PubMed® and the Web of Science^TM^ databases were searched in January and February 2022 for original mouse research reports published between 01/2018 and 02/2022; focused on monotrauma involving the head, bone, abdomen, and thorax or monotrauma in combination with hemorrhagic shock (polytrauma); involved murine models that represent improvements in clinical relevancy compared to previous model versions; and provided clear methods to enhance reproducibility.

## Reconsideration of Murine Animal Models in the Immunological Trauma Research

Generally, mice are the animal model of choice to investigate underlying mechanisms in diseases or various immunological settings and are responsible for important breakthroughs in understanding the human immune system. Reasons are cost-effective keeping, easy implementation, ethically acceptance by the public, genetic manipulation in the form of knockout models as well as high gene homology between mice and human (approximately 80%) (Tsukamoto and Pape 2009).

However, it needs to be mentioned that criticism surrounds murine models with regards to translational research. In this context, the “Inflammation and Host Response to Injury” (Xiao et al., 2011; Cuschieri et al., 2012) as well as the Mouse ENCODE Consortium (Yue et al., 2014) listed the response of mice and humans on the transcriptomic level. These datasets have been assessed and evaluated by separate working groups, resulting in differing conclusions with regards to the genomic response to inflammatory settings (Seok et al., 2013; Takao and Miyakawa 2015).

The choice of the mouse strain also seems to be an issue for trauma research as different strains might exhibit different properties, relevant for the trauma response. First, different inbred (e.g., C57BL/6) and outbred (e.g., ICR) strains are available. While inbred strains show a high genetic stability and therefore potentially increase experimental reproducibility, outbred strains are genetically heterogeneous and therefore better simulate the human population (Tuttle et al., 2018; Spenlingwimmer et al., 2019) thereby presenting an important factor when establishing clinically relevant models. Moreover, Tuttle et al. could not find evidence for greater trait stability in inbred mice compared to outbred mice and therefore suggested the use of outbred mice for biomedical research (Tuttle et al., 2018). However, mouse strains not only differ regarding their breeding method but also in physiological properties like heart and skeletal muscle masses (Avila et al. 2017) and their immune response to injury or infection. In a sepsis model, BALB/c mice were shown to respond in a Th2-dominant manner compared to C57BL/6 mice, which respond in a Th-1 dominant manner, probably caused by different innate immune response of macrophages (Watanabe et al., 2004). Furthermore, the airways response to injury in C57BL/6 was comparable to that in outbred strains and humans, which was not the case for BALB/c mice (Busch et al., 2016). Additionally, BALB/c mice showed higher levels of circulating regulatory T-cells and MHC-2-positive lymphocytes compared to CD-1 mice in response to polytrauma, whereas overall immune response was comparable (Spenlingwimmer et al., 2019). With regard to trauma-hemorrhage C57BL/6 mice showed significant differences in splenocyte and bone-marrow functions as well as in release of immune mediators compared to C3H/HeN mice (Matsutani et al., 2005).

Apart from that, one issue often discussed in literature concerns the age of the mice that are used in experiments as it influences the immune system, organ function as well as metabolites (Nikolich-Žugich 2014; Petr et al., 2021). As shown in Table 1, all but one (pediatric study) of the mentioned studies used mice within the age of 8–17 weeks representing the age range most often used for murine models, although the term “adult” seems to be inconsistently defined across studies (Jackson et al., 2017). For C57BL/6J mice, three life phases, namely mature adult (3–6 months corresponds to 20–30 years in human), middle-aged (10–14 months corresponds to 38–47 years in human), and old (18–24 months corresponds to 56–69 years in human) are described (Flurkey et al., 2007). The use of mature adult mice has several biological advantages as they are considered as fully developed but not yet affected by senescence, although one of the main reasons seemed to be reduced costs as well as historical data comparability (Jackson et al., 2017). However, the gold standard for age might not exist and should depend on the scientific question; for example in some mouse strains the peak bone mass is not reached within the age span of 3–6 months (Jilka 2013). Obviously, the age of the human counterpart should always serve as a reference.

Furthermore, it is criticized that one of the advantages of murine trauma models namely the controlled environment is limiting research with regards to underlying inflammatory processes and mechanisms due to the missing multifactorial nature of a traumatic injury. All models listed in Table 1 use mechanical, blunt trauma induction with the intention to create a reproducible, multifactorial nature of trauma, imitating the course of injury (accident, fall, hit, explosion, bone fracture) in a best possible way and overcoming the limitations existing for injuries induced *via* chemical induction like bleomycin-induced lung injury (Orlando et al., 2019), BaCl_2_-induced muscle injury (Hardy et al., 2016) or glutamate-induced spinal cord injury (Cheriyan et al., 2014) more relevant for a highly controlled injury induction.

Additionally, no drug or therapeutic reagent that showed success to some degree in murine trauma models was able to show comparable results in clinical trials (Efron et al., 2015; Stortz et al., 2017; Mira et al., 2018). Therefore, an alternative approach described as “reverse translation” (Efron et al., 2015) was proposed and should be taken into account when establishing new trauma models. It requires the verification of the murine model based on observations (specific molecules, phenotypes) made in the clinic and simplifies the final retranslation to the patient. Due to the development of high-resolution methods like next generation sequencing, single-cell RNA-Seq or mass cytometry but also the progress made in imaging techniques the data situation in humans improved within recent years making this approach even more applicable. On the other hand, these methods should be also applied in the respective murine model to confirm and verify its suitability. Although associated with higher effort, costs and need of expertise, we recommend using the “reverse translation” approach for the development of murine models as it might greatly enhance the translatability of murine research to human.

## Discussion

After reviewing various aspects that clearly indicate some disadvantages of murine models for investigating the immune response to multiple trauma, the questions arise if these models should be replaced in trauma research. Even though no clinical success was achieved with mouse models in trauma research, most innovations in human research were based on mouse models, due to the unethically nature of immediate human trials (Mira et al., 2018; Efron et al., 2015; Stortz et al., 2017). Furthermore, mouse models have proven themselves as an important tool in other translational research fields including immunology (Osuchowski et al., 2014). The solution should be to revise existing models instead of abandoning them. Each of the presented trauma models in Table 1 was chosen because it added some modification to an established trauma model to get closer towards the human situation. Either by adding more trauma to resemble the multifactorial side of a human trauma or by adding specific ways to set a trauma to be more consistent with the circumstances of human trauma (brain contusion by explosion modeled through blast wave). Clinical relevance should always be the center point of the murine mouse model. If the main population of patients suffering multiple trauma are adults, there is no need to set up a model with 10-week-old mice (Wang et al., 2020), although several advantages are associated with this age range. If blunt trauma is investigated, chemical induction might not be the best choice due to a different nature of the trauma.

In conclusion, we are of the opinion that the represented models serve as a good starting point for further development of animal models under consideration of the “reverse translational” approach, keeping the focus on the important aspect of clinical relevance and clinical translatability. As this approach requires a profound knowledge about the complex trauma model, the investigation of the respective monotrauma should always be included although, at first sight, increasing effort and animal numbers. Nevertheless, a well investigated and described model proven to be reliable and clinically relevant enhances reproducibility and has the potential to become a standard model in this research field simultaneously compensating effort and animal numbers.

When developing models with the intention to describe a molecular mechanism, the underlying trauma model should be proven to be clinically relevant as described before. Within their work, researchers should clearly state whether and how the used model addresses a clinically relevant situation, intends to elucidate a molecular mechanism, or even tries to combine both aspects. However, the downsides of using murine models in such a complex field of research, as represented within this review, should always be borne in mind. Apart from focusing on clinical relevance and clinical translatability, further improvement of existing models should also consider the importance of social determinants of health as they are relevant for the outcome and mortality after trauma (Phelos et al., 2022), but are often overlooked within current studies.
